# Partial Genome Sequences of Human Norovirus Strains from Northeast Brazil

**DOI:** 10.1128/MRA.01135-19

**Published:** 2020-01-02

**Authors:** Klarissa Miranda Guarines, Renata Pêssoa Germano Mendes, Jurandy Júnior Ferraz de Magalhães, Lindomar Pena

**Affiliations:** a Department of Virology, Aggeu Magalhães Institute (IAM), Oswaldo Cruz Foundation (Fiocruz), Recife, Pernambuco, Brazil; b Pernambuco State Central Laboratory (LACEN/PE), Department of Virology, Recife, Pernambuco, Brazil; KU Leuven

## Abstract

Noroviruses are the leading cause of human gastroenteritis worldwide. Here, we sequenced the open reading frame 1 (ORF1)-ORF2 junction region of norovirus strains isolated from 20 human stool samples. Samples were collected between 2014 and 2017 in Pernambuco State, Brazil. Phylogenetic analyses identified four norovirus GII genotypes circulating in this area of the country.

## ANNOUNCEMENT

Norovirus (NoV) is the main cause of nonbacterial acute gastroenteritis (AGE) worldwide (1). In Latin America, NoV represents 15% of all AGE outbreaks (2). In Brazil, NoV has received greater attention since the introduction of national rotavirus vaccination (3). The virus is highly contagious and spreads mainly by direct contact from person to person (4). The *Norovirus* genus belongs to the *Caliciviridae* family and has a positive single-stranded RNA genome ∼7.5 kb long, typically organized in three open reading frames (ORFs). ORF1 encodes nonstructural proteins, including the RNA-dependent RNA polymerase (RdRp), ORF2 encodes the major capsid protein VP1, and ORF3 encodes the minor capsid protein VP2.

NoV can be classified into 10 genogroups (GI through GX) and further subclassified into 49 genotypes based on phylogenetic analysis of the RdRp and the *vp1* gene (5). Genogroups GI, GII, and GIV cause disease in humans, and the most commonly detected genotype is GII.4 (6, 7). Here, we report, for the first time, the sequencing and molecular characterization of NoV identified in Pernambuco State, Northeast Brazil. Twenty human stool samples were selected from NoV-positive patients previously diagnosed by Pernambuco State Central Laboratory (LACEN/PE) between 2014 and 2017. The selection of samples for sequencing was based on the year of collection and the quality of amplification seen with agarose gel electrophoresis analysis. Samples were derived from patients who had AGE symptoms, and their ages ranged from 2 months to 2 years.

Viral RNA was extracted from 140 μl of fecal suspension with the QIAamp viral RNA minikit (Qiagen, CA, USA) according to the manufacturer’s instructions. For sequencing, RNA was first reverse transcribed into complementary DNA (cDNA) using the ImProm-II reverse transcription system (Promega, USA). cDNA was amplified using GoTaq Green Master Mix (Promega, USA) and the primers 11F (AGTGGAATTCCATCGCCCACTGG) and 11R (GGCTTGGACAAAATTTGTTCTAATCCAGGG), targeting a 786-bp region in the ORF1-ORF2 junction. Amplified products were purified with the QIAquick PCR purification kit (Qiagen, USA). Samples were sequenced in both directions with a BigDye direct Sanger sequencing kit (Thermo Fisher Scientific, Waltham, MA, USA) in the ABI Prism 3730 genetic analyzer (Applied Biosystems, Foster City, CA, USA).

**FIG 1:**
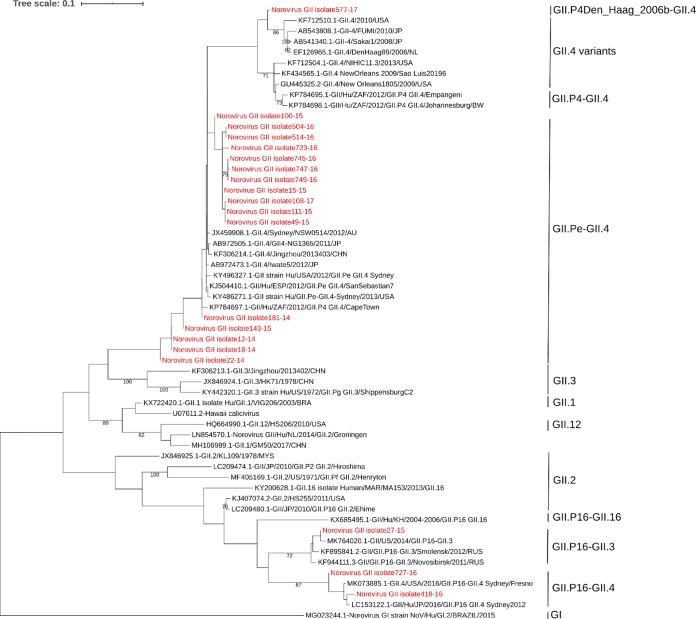
Phylogenetic tree. The novel norovirus GII strains (*n* = 20) isolated in this study are indicated in the phylogenetic analysis in red. Reference strains were downloaded from GenBank. Norovirus GI (accession number MG023244) was used as the outgroup. Sequence multiple alignments were performed using the ClustalW algorithm. The maximum likelihood phylogenetic tree was constructed with MEGA X software with bootstrap tests (1,000 replicates) and edited on iTOL Tree (https://itol.embl.de/). The bootstrap percentage values of ≥70% are shown at each branch point.

Consensus sequences were aligned and edited with the SeqMan DNAStar Lasergene package (DNAStar, WI, USA). The lengths of obtained sequences varied from 315 to 730 bp because of the variable quality of the obtained Sanger reads. Genotyping was performed using the Norovirus Typing Tool version 2.0 (8). Multiple alignments were performed with ClustalW. A phylogenetic tree was constructed using the maximum likelihood method and the Tamura-Nei model using MEGA X software with default parameters (9, 10) (Fig. 1).

Four norovirus GII genotypes (GII.Pe-GII.4, *n* = 16; GII.P16-GII.3, *n* = 1; GII.P16-GII.4, *n* = 2; GII.P4-GII.4, *n* = 1) were found to be circulating in this region of the country. These genotypes were closely related to the following strains: GII.4 strain DenHaag89/2006/NL, GII strain Hu/USA/2012/GII.Pe-GII.4 Sydney, GII strain US/2014/GII.P16-GII.3, and GII strain Hu/JP/2016/GII.P16-GII.4 Sydney2012. In summary, our study describes the genetic characterization of 20 NoV strains from Northeast Brazil and sheds light on the molecular epidemiology of NoV in the tropical world.

### Data availability.

The GenBank accession numbers for the norovirus genome sequences are MN416493 to MN416512.
